# Fecal Sludge Management in Low Income Settlements: Case Study of Nakuru, Kenya

**DOI:** 10.3389/fpubh.2021.750309

**Published:** 2021-10-11

**Authors:** Sheillah Simiyu, Ivy Chumo, Blessing Mberu

**Affiliations:** Urbanization and Well-Being Unit, African Population and Health Research Center, Nairobi, Kenya

**Keywords:** fecal sludge management, low income settlements, Nakuru, safely managed sanitation, sanitation value chain

## Abstract

**Introduction:** In order to meet the sustainable development goals targets of sanitation, countries aim to increase access to safely managed sanitation services for its citizens. Safely managed sanitation services refers to improved sanitation technologies that are not shared with other households and where excreta is treated and disposed; or stored, transported and treated off-site. In most Sub-Saharan Africa (SSA) countries, on-site sanitation facilities such as latrines and septic tanks are common, with low-income urban settlements mainly using pit latrines. However, little is documented about the management of sludge from these facilities, especially in low income settlements in secondary and emerging cities. This lack of data is a major hindrance to public health, development and planning efforts by governments and planning agencies. This study specifically assesses practices and challenges along the sanitation value chain related to containment, emptying, transportation, treatment and recycling of fecal sludge.

**Methods:** The study was carried out in low income settlements in Nakuru, a secondary city in Kenya. Over half the population in Nakuru live in low income areas and majority of these residents use pit latrines. A case study design was selected for this study and data was collected using qualitative methods. Data was collected through In-depth interviews and Focus Group Discussions using in depth interview guide and focus group discussion guides that had questions on sanitation practices along the value chain, challenges, opportunities available, and recommendations for improvement. Analysis was done through content analysis by reading the transcripts multiple times to gain a sense of the flow of the discussion. Thereafter, coding was done by following emergent issues and thereafter categories were identified which formed the basis for providing a picture of FWM practices in the settlements.

**Results:** On site sanitation facilities are dominant in the settlements, but they are few and are shared by several households. These facilities were unclean, and they filled up at a fast rate because of the high number of users. The latrines were emptied by manual emptiers who used mechanized equipment but complemented with manual emptying using buckets. Sludge was transported to a central collection point using large and small scale means of transportation, before transfer to the treatment site for final treatment and disposal. Various stakeholders are involved in capacity building of emptiers as well as in the transportation, treatment and disposal of fecal sludge in the settlements. Challenges along the stages of the value chain included negative community perceptions and attitudes toward fecal sludge management.

**Conclusion:** The results highlight the need to address the challenges along the chain by involvement of state and non-state actors. Low income areas have high populations and thus contribute huge amounts of fecal sludge. Deliberate efforts to consolidate such data from low income areas will result in availability of data, and informed decision making for stakeholders at national and international levels.

## Introduction

The sixth SDG aims to “ensure availability and sustainable management of water and sanitation for all” and targets “adequate and equitable sanitation and hygiene for all, ending open defecation, and paying special attention to the needs of women and those in vulnerable situations.” One of the indicators for measuring this target is the proportion of the population using “safely managed sanitation services” (1). “Safely managed sanitation services” refers to improved sanitation technologies that are not shared with other households and where excreta is treated and disposed; or stored, transported and treated off-site (2). As such, safely managed sanitation services include sewer systems where wastewater is treated at treatment plants, latrines whose excreta is removed and treated at designated sites, and sanitation technologies where excreta is treated and disposed of *in situ* (e.g., in septic tanks) (2). In most Sub-Saharan Africa (SSA) countries, on-site sanitation facilities such as latrines and septic tanks are common than sewer systems (2) and the use of these on-site facilities varies considerably within countries, with low-income urban settlements mainly using pit latrines (3). When these latrines fill up, households have to empty them because of the lack of space to dig new pits. Safe management of sanitation services in such settlements requires emptying, transportation, treatment and disposal or recycling of the sludge from these latrines.

By 2017, ~29% of Kenya's population had basic sanitation (facilities that are not shared with other households) and only 5% had access to the sewer system (1). Approximately 20% of the population in urban areas use toilets connected to the sewer system, and 47% use pit latrines (1). In the capital city of Nairobi for example, over half of the population lives in low income settlements (4) where residents mainly use on-site sanitation facilities and/or open disposal of fecal waste (5). In these settlements, interventions that aim at safe containment of fecal sludge such as communal and portable sanitation facilities (6–8) have been introduced; and studies have been implemented to test the feasibility of treatment of fecal sludge (9).

Considerable research has been done in primary cities, but very little is known about secondary cities in SSA. Population is booming in Africa's secondary cities which link remote and rural areas to urban centers, and provide alternative economic opportunities to public services and facilities. The devolved governance system in Kenya for instance, established county headquarters across the country, which have emerged as new centers of counter attraction for migrants from other rural and urban areas. This growth has created questions around the provision of critical infrastructure and services such as water and sanitation for the rapidly expanding secondary cities, especially among the most vulnerable segments of residents who live in low income settlements.

**Figure 1:**
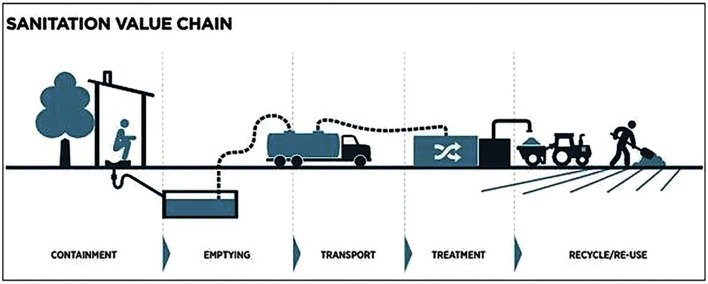
The sanitation value chain. Source: BMGF, 2012 (11).

The Kenyan government singles out the need to focus on non-sewered sanitation in urban areas and on fecal sludge management through the full sanitation value chain, as measures to meet the sanitation goals (10). Meeting these national targets and achieving the global targets of adequacy and equity in sanitation requires that interventions be expanded to the growing and emerging secondary cities. These interventions should be aligned with the prevailing conditions, and/or build upon already existing interventions/efforts. Few studies, however, have documented or detailed these prevailing conditions, especially with regards to fecal sludge management in low income settlements from secondary cities in Kenya. In fact, lack of local level data across African cities is a hindrance to programming; yet such data enables identifying priorities and interventions that work and measuring progress. This paper, therefore, aims to provide a situational analysis of fecal sludge management practices in low income settlements of Nakuru town in Kenya. The study specifically assesses practices and challenges along the sanitation value chain (Figure 1) related to containment, emptying, transportation, treatment and recycling of fecal sludge.

## Methods

### Study Area

Nakuru town is the headquarters of Nakuru County, and is one of the rapidly expanding counties in Kenya. By 2019, the County's population was 2.2 million- an increase from 1.6 million in 2009 (12). Approximately half the population is aged below 20 years and about 75% aged below 30 years (12).

Nakuru is the fourth largest urban center in Kenya and by 2019, the city had a population of ~400,000 persons (12). The town is administratively divided into Nakuru Town East and Nakuru Town West constituencies (13). Over half the population in Nakuru live in low income areas (LIAs) which include Bondeni, Manyani, Kaptembwo, Flamingo, Kapkures, Rhonda, and Gituima (13). Living conditions in these areas are characteristic of other low income settlements in Kenya and include poor quality housing, lack of/poor provision of services such as water and sanitation, and poor solid waste management services (13). With regards to sanitation, majority of the residents in the low income areas mainly use pit latrines (13, 14).

There are several state and non-state actors involved in sanitation service provision in Nakuru town. The Nakuru County Government, like other county governments, is responsible for policy formulation, resource mobilization, service provision and policy implementation. The Nakuru Water and Sanitation Services Company (NAWASSCO) is the main water utility responsible for water and sanitation service provision in the County. Non-governmental organizations such as Water and Sanitation for the urban poor (WSUP) are involved in interventions aimed at improving access to sanitation in the county, and they work with several Community based groups. In 2019, the Nakuru Countywide Inclusive Sanitation Strategy and the Nakuru Countywide Sanitation Steering Committee (NACOSTEC) were launched. The strategy provides a framework for improving sanitation for all in the county and the steering committee co-ordinates the implementation of sanitation activities in the county.

### Sampling Procedures

A case study design was selected for this study and data was collected using qualitative methods. For representativeness, low income settlements were randomly selected from Nakuru Town East and Nakuru Town West constituencies. The study was conducted in Flamingo, Kivumbini, and Biashara settlements (in Nakuru Town East Constituency); and Kaptembwo, Rhonda, and Kapkures settlements in Nakuru Town West Constituency.

Different sampling strategies were used to select respondents involved in fecal sludge management along the sanitation value chain. Respondents were selected from the two constituencies as participants of In-depth Interviews (IDIs) or Focus Group Discussions (FGDs). IDIs were conducted with women and youth leaders, village heads, administrative leaders (chiefs/assistant chief), manual pit emptiers, and service providers at the treatment plant. Two women and youth leaders were selected from each of the constituencies to represent views from the settlements within their constituencies. Additionally, two village heads (a male and female) who had lived within the settlements for averagely 10 years were selected to be included in the study, and one chief/assistant chief was purposely selected from each of the settlements. There were only two groups of organized manual emptiers at the time of the study, with one group operating in Nakuru Town East and another in Nakuru Town West; and thus one leader, who had served the longest, was selected from each of the groups. Some pit emptiers were not in any organized group, and since they were difficult to trace, snowballing was used to identify them if they worked in the settlements in Nakuru Town East and Nakuru Town West constituencies. Sanitation service providers along the sanitation service chain were purposively selected if they had been involved in service provision since the inception of such programs.

FGDs were conducted with women, youth, and men groups; and manual pit emptiers. To select FGD participants, the men, women, and youth groups involved in sanitation related activities were identified from the two constituencies. Two groups of each of these categories were identified and included in the study. Participants were selected if they were members of these groups. Similarly, members from the two groups of organized manual pit emptiers were invited to participate in FGDs.

### Data Collection

The interviews were guided by an in-depth interview guide which had questions on sanitation practices along the value chain, challenges, opportunities available, and recommendations for improvement. Interviews were conducted in English and/or Swahili, and were held at a location convenient and comfortable to the participants (mostly in a quiet community hall or at the participants' workplace). These interviews were conducted by trained research assistants who were accompanied by the researchers. The interviews were recorded using voice recorders and backed up with notes that were written in note books by the assistants. The interviews lasted for a maximum of 1 h. Selection and interviewing of participants continued until the point when new information was not forthcoming.

Data was also collected through FGDs to complement the information obtained from the IDIs, and also since FGDs are useful in collecting information from people like pit emptiers who may not be easily reachable and/or may not readily divulge information through interviews. The FGDs had ~8–12 participants and were mainly held at community halls. The FGDs were conducted by a team composed of a moderator, a note taker and a team leader. The moderator guided the discussion, the note taker took notes and observed any non-verbal cues, and the team leader's role was to oversee and trouble shoot any problems, clarify any issues or questions from the research assistants, and perform spot checks to enhance quality of data. The discussions were guided by a guide that had questions on sanitation practices along the value chain, sanitation challenges, and opportunities and recommendations for improvement. Information was captured using voice recorders and supplemented by notes taken in notebooks. The discussions lasted for at least 1 h.

### Data Quality Control

Research assistants were selected if they had been endorsed by community leaders in the study sites, and if they had at least 5 years' experience in qualitative research. The assistants were trained for 5 days on the aims of the study, data collection process, data collection tools, and research ethics. The tools were pre-tested in Kaptembwo and Flamingo areas for 2 days using the local language. FGD guides were pre-tested with youth and men and IDI guides were pre-tested with women and a village head; all of whom who were not included in the study. The aim of the pre-testing was to assess if the questions on the tools were well-understood by both research assistants and interview participants, and ensure that any emerging questions were captured. After the pre-test, the tools were further refined to capture all emergent issues. During fieldwork, field supervisors accompanied the research teams to ensure that probing was done correctly and to assess any threats to data quality. Debriefing sessions were held at the end of each working day to highlight the major findings, to review effectiveness of probing techniques, and to assess progress.

### Ethical Considerations

Ethical approval was obtained from AMREF Health Africa's Ethics & Scientific Review committee (ESRC), Ref No: AMREF-ESRC P375/2017 and also from National Commission for Science, Technology and Innovation (NACOSTI), Ref No: NACOSTI/P/17/99937/20578. Approval was also obtained from all the relevant authorities at county and community levels before commencement of study activities. Each participant received a research information sheet before the interview. The sheet contained information about the purpose of the study, study procedures, how the data would be utilized; institutional affiliation of the researchers; the right to withdraw at any time without reprisal; and measures to ensure confidentiality of information. The interviewer explained the contents of the information sheet to the participants and gave them an opportunity to ask any questions. Thereafter, participants signed a consent form if they voluntarily agreed to participate in the research. The recordings and transcripts were stored in a password protected computer that was only accessible to the investigators, all of whom signed a confidentiality agreement.

### Data Management and Analysis

Data analysis began during data collection and continued until data collection was complete. After data collection, researchers listened to the recordings again to familiarize themselves with the information. The recordings were then transcribed into MS Word, and cross checked by a third party to ensure that all the information had been captured in the transcript. The transcripts were translated to English (where necessary) and again cross checked to ensure that the translation did not alter the meanings of the data. After translation, the transcripts were imported into NVIVO 10 software (QSR International, Australia) for coding and analysis. Each transcript had a unique identifier comprising of date and participant identifier to enhance anonymity and facilitate informed analysis. Analysis was done using standard methods of content analysis by reading the transcripts multiple times to gain a sense of the flow of the discussion. Thereafter, coding was done by following emergent issues (codes) such as sanitation facilities, emptying process, transportation process etc. This coding was applied to all transcripts and as new issues emerged, they were added to the list of codes and the transcripts read again to ensure that all the transcripts had been coded adequately. Issues that were similar were combined into single categories through consensus discussions. The next step entailed producing “tree nodes” or major categories that were inductively synthesized from the previous steps. The categories identified formed the basis for providing a robust picture of FWM practices in the settlements.

## Results

A total of 14 FGDs were held with emptiers, women groups, youth groups and men leaders in the community, and 36 IDIs were conducted with emptiers, technical persons in the recycling plants, chiefs/assistant chiefs, village heads, youth and women leaders. This information is summarized in Table 1. Results will be presented according to the stages of the sanitation value chain; i.e., containment, emptying, transportation and treatment and disposal. Each of the sections will highlight the practices and challenges faced.

**Table 1:** Distribution of study participants and data collection methods.

**Method**	**Participants**	**Number**
FGDs	Emptiers	2
	Women groups	4
	Youth groups	4
	Men groups	4
	Total	14
IDIs	Emptiers	6
	Service providers and technical persons	4
	Chiefs/Assistant Chiefs	6
	Village heads	12
	Youth leaders	4
	Women leaders	4
	Total	36

### Containment

Residents made use of both onsite and offsite sanitation technologies. Onsite sanitation technologies included toilets connected to septic tanks and pit latrines, while offsite technologies included flush toilets connected to the sewer system. Most of the residents, however, used pit latrines whose construction costs were paid by plot owners. Due to a high population density in the settlements, these sanitation facilities were few and were usually shared among households, with respondents reporting high numbers of users sharing the available latrines.

*“We are sharing toilets, sometimes 100 people in the plot sharing 4 toilets….”* (FGD, Female, Nakuru Town East).

Participants further noted that these latrines were not clean, and women particularly were concerned about the effects of unclean toilets. Participants also complained of foul odor from the toilets due to the high number of users and poor cleaning practices. Due to this high number of users and the smelly and dirty toilets, participants admitted that they used other alternatives such as defecating in bags which were then disposed in the open spaces. Children's feces were also disposed in open spaces.

With regard to structural attributes, the latrines had unlined and shallow pits, and were easily prone to collapse. Due to the high number of users and the shallow depths of the pits, the latrines filled up at a fast rate. Participants reported that when residents did not have money to pay for emptying, they poured a chemical substance that was believed to absorb the liquid in the pit, thereby reducing the volume of the sludge.

*“Sometimes when there is no ready money for emptying, we buy a (salt- like) traditional substance and pour into the pits to prevent the pits from filling up fast.”* (FGD, Male, Nakuru East).

### Emptying

Emptying was done by manual pit emptiers using buckets or by using emptying equipment (gulper and Rama). These emptiers were organized in groups, and sometimes engaged themselves in other community activities such as solid waste management. Interviews with NAWASSCO staff revealed that the emptiers initially worked in the night and disposed the sludge in an unhygienic manner. NAWASSCO identified these emptiers, trained them on emptying techniques, and provided them with protective gear (gloves, goggles, masks, and overalls), disinfectant, and sprays. After training, the emptiers were given certificates and mechanized emptying equipment-the Gulper and Rama to complement manual emptying.

According to the pit emptiers, lined pit latrines were usually emptied mechanically using the gulper and the Rama. However, since the equipment could not empty beyond a certain depth (thus unable to empty all the sludge from the pits), they complemented with manual emptying. In addition, the emptiers noted that pit latrines had a lot of solid waste, making emptying difficult and necessitating manual emptying.

*“…you will find a cloth… a blanket… recently we found a human head inside the pit latrine… so before you begin emptying you start sorting and removing the solid waste…”* (IDI Emptier Nakuru Town West).

Unlined pit latrines were usually emptied manually mainly because they were prone to caving in during emptying because of poor construction.

Despite having been given protective gear, emptiers admitted that they sometimes worked without any protective gear, or they used tattered gear because of worn out gear. The emptiers, however, understood the risk of not using protective gear, as they admitted that working without protective equipment “*can make one sick*” (FGD, Pit Emptiers, Nakuru Town West).

Pit emptying costs varied between KES 1500 and KES 3000. The cost was often subject to negotiations and was determined by the size of the pit, the amount of sludge in the pit, the weather season (rainy seasons were cheaper because of availability of water, while dry seasons entailed additional cost of purchasing water to dilute the sludge), and whether the pit latrine was lined or unlined (lined pits were cheaper due to less risks compared to unlined pits). Recipients of the emptying service paid for the costs of emptying. Often times, tenants shared the costs of emptying among themselves.

Pit emptiers highlighted that some landlords, especially the elderly who relied on rental income from the rental units they owned, did not pay in time, they paid less, or they did not pay at all. Due to the low proceeds from their services, caused by delays or non-payment, and the fact that emptying was not usually done every day; emptiers engaged themselves in other businesses like motorcycle riding or unblocking sewers to earn income when pit emptying work was minimal.

Evidently, pit emptiers faced stigma from community members. An FGD participant for example, expressed her dislike for the emptiers by saying that

*“…They* [pit emptiers] *are dirty…they inhale dirty things while working and I would not engage them in my day to day activities”* (FGD, Female, Nakuru Town East).

Due to such humiliation from community members, the emptiers continued emptying the pit latrines at night, and others reportedly worked under the influence of alcohol and/or drugs despite efforts from the municipal council and public health officers to empower them against alcohol and drugs. The emptiers admitted that they engaged in drugs and alcohol as a way of disconnecting themselves from the stigma.

*“At times I have to use alcohol…so that I don't pay attention to comments from people… young women laugh at us…. They ask us who we shall marry …. If you are sober you can give up…*.” (IDI, Manual pit emptier, Nakuru Town West).

### Transportation

After emptying, the waste was transferred to Primary Collection Points (PCPs). PCPs are 10 cubic meter-tanks distributed by NAWASSCO and erected at selected points in the community to facilitate collection of sludge from different points. These sites were selected after consultations with community members and the pit emptiers. When the tanks were full, sludge was picked by NAWASSCO staff and transported to the treatment plant.

Private transport facilities were mostly used to transport sludge to the treatment plant because the county government had limited transportation services. Sludge was transported on small and large scale. Small scale transportation made use of buckets and wheel carts while large scale transportation used trucks. A few trucks were owned by the county government but majority of the trucks were privately owned. Because of the business opportunity presented by transportation of sludge, private individuals had invested in exhaust trucks to complement the few trucks from the County government. These private trucks were all registered by the County government, and they had a permit while awaiting licensing from NAWASSCO. Acquiring a means of transport from the County government was reportedly slow and challenging. According to the emptiers, the application process was tedious mainly because of bureaucracy. Additionally, emptiers reported that trucks were expensive to hire, and thus, they mainly transported sludge using wheel carts which were slow and which contributed to spillage of the sludge during transportation. Spillage also occurred due to untarmacked and hilly roads, as well as through cracks from some transportation trucks.

### Disposal, Treatment, and Recycling

The water utility company, NAWASSCO, was involved in large scale treatment of sludge. The treatment plant at NAWASSCO had been expanded (and set up as a prototype) to include recycling and reuse of fecal sludge. As a result, the company had begun the production of briquettes.

At the plant, sludge was burnt under high temperature to kill microorganisms and clear foul odor, and was then taken for further processing and recycling into briquettes. The briquettes were then sold to community members and members of the public at KES 30 per kilogram. Staff at NAWASSCO highlighted that they had produced enough briquettes since the demand was more than the supply. The company hoped to acquire more mechanized equipment to enhance efficiency in the production of briquettes. They however noted that dust and smoke emissions were a challenge, despite efforts to minimize the emissions.

Community members were trained by NAWASSCO on how to use briquettes during monthly meetings with the County Government and the community. Community members were thus aware of the advantages of sludge as a source of income, and the use of briquettes. FGD participants, however, complained that they had not been educated on how the briquettes were made, as well as their negative impacts.

Generally, although fecal sludge management presented an employment opportunity, few people were aware of the available opportunities. Perceptions of recycling of fecal sludge varied, as some individuals had negative perceptions about the opportunities provided by fecal sludge. A female worker who was involved in recycling for example noted that

*“Not many people are aware of the jobs that come with fecal waste… many people in the community critique us for doing this job … and especially being a woman…”* (IDI with a recycler, Nakuru Town West).

## Discussion

This study has detailed fecal sludge management practices in low income settlements in Nakuru town in Kenya. On site sanitation facilities are dominant in the settlements, but they are few and are shared by several households. These facilities were unclean, and they filled up at a fast rate because of the high number of users. The latrines were emptied by manual emptiers who used mechanized equipment but complemented with manual emptying using buckets. Sludge was transported to a central collection point using large and small scale means of transportation, before transfer to the treatment site for final treatment and disposal. NAWASSCO and other stakeholders are involved in capacity building of emptiers as well as in the transportation, treatment and disposal of fecal sludge in the settlements. Various challenges along the stages of the value chain were evident, including negative community perceptions and attitudes toward fecal sludge management.

The dominance of on-site sanitation facilities in the settlements was not surprising as literature has alluded to the high investment and operation costs of the sewer system and lower operational and maintenance costs of onsite sanitation facilities (15, 16). Such high costs imply that the sewer system is non-existent in many low income settlements. In addition, the available on-site sanitation facilities are often shared among households in low income settlements due to the challenges of lack of space to construct sanitation facilities (17). Sharing presents various challenges and leads to a faster fill up of the pit latrines, as was evident in our study.

Due to space limitations, emptying is the most appropriate option for full latrines in low income settlements. Pit latrine emptying in Nakuru was akin to other cities in Africa (18, 19) where varied mechanized and manual technologies are used for pit latrine emptying (20, 21) and often by manual emptiers. These private emptiers are an alternative to emptying services provided by the Ministry/service authority, which are often times inadequate to meet the demand, or are hindered by the lack of space in the settlements which prevents access of emptying trucks. Research from other low income settlements in Kenya suggests that these private emptiers, who are often from the community, are usually preferred, because of their ability to navigate the narrow spaces in the settlements, their ability to empty solids from the pits, and they have flexible payment plans favorable for households. When the private sector is involved in service provision, customers are able to negotiate the price, while the service providers determine the price based on other factors such as the size and contents of the pit, and the relationship they have with the clients (22, 23). Nonetheless, results from this study points to the safety and acceptability of the manual emptiers as an area of intervention, especially with regards to the use of protective gear and stigma from community members. Although NAWASSCO has built the capacity of these workers, findings show the need for community sensitization, regular refresher courses, and provision of protective gear to enable effective service delivery from the emptiers.

A highlight of the study was the citing of primary collection points where sludge is contained before final transportation to the treatment and disposal site. Transportation of sludge is costly, and depends on factors such as distance and technology used for transportation (24, 25). As such, since households often pay for emptying, the costs of transportation often fall onto the households thereby increasing the costs of emptying. The location of collection points close to the settlements reduces the distance traveled by emptiers thereby minimizing the chances of indiscriminate disposal of sludge in the environment (24).

In Nakuru, the production of briquettes from fecal sludge was an avenue for business, resource recovery and reduction of environmental contamination from pathogens and heavy metals contained in fecal sludge (26, 27). Apart from briquettes as an energy source, fecal sludge has the potential for recovery of biogas, and can be used as a soil conditioner and in building materials (28, 29). These are opportunities for further use of the recycled sludge, calling for an assessments of the viability of these opportunities, of the effects of emissions released into the environment (30), and a reduction of these emissions. Continued sensitization on the benefits of recycled sludge products is also needed through the use of the community leaders and stakeholders in the settlements.

These results show that achieving sustainable sanitation in the low income settlements requires the involvement of various stakeholders along the value chain, including community members, the private sector, and the relevant authorities. The community are end users of the recycled products, and need to manage their sanitation facilities, while the private and municipal sector facilitate emptying, transportation and treatment of sludge. These stakeholders need to work in tandem, to ensure achievement of the sanitation goals. By so doing, sustainable sanitation will contribute to sustainable urban development and the achievement of SDG targets (31). Proper fecal sludge management in such settlements contributes to achieving the SDG targets for the population living in secondary cities and in achieving the global sanitation goals.

We acknowledge however that this study was mainly qualitative, and is only limited to the low income settlements. Further studies, to collect quantitative data are needed to complement the information obtained in our study. Such information should also be used to complement available data on fecal sludge management in Nakuru town and Nakuru county as a whole, including updating Nakuru's shit flow diagram (17).

## Conclusion and Recommendations

This study sought to provide a situational analysis of practices and challenges related to containment, emptying, transportation, treatment and recycling of fecal sludge in low income settlements of Nakuru, a rapidly expanding town in Kenya. Following a lack of local context data from such towns to inform interventions and planning, this paper provides a qualitative assessment of fecal sludge management in the settlements, and notes the challenges of effective sludge management in low income settlements of growing towns in developing countries. The highlighted challenges serve as entry points to effective fecal sludge management in these settlements, thereby contributing to national and global tracking systems as well as planning and monitoring initiatives. Our study highlights the need for quantifying the amount of fecal sludge that is safely managed in the settlements, and comparing with data from the county level. The settlements host a majority of onsite facilities and a citywide assessment of the management of fecal sludge is necessary and will be useful for planning and monitoring of the country and global goals. Finally, this study points to possible areas of intervention; such as the safety and health of manual emptiers, and the possibility of other resources as by-products from of fecal sludge.

## Data Availability Statement

The original contributions presented in the study are included in the article/supplementary material, further inquiries can be directed to the corresponding author/s.

## Ethics Statement

The studies involving human participants were reviewed and approved by the Ethics and Scientific Review Committee of Amref Health Africa. The patients/participants provided their written informed consent to participate in this study.

## Author Contributions

SS wrote and revised the manuscript. IC supervised data collection and participated in manuscript writing and analysis. BM conceived the idea, provided overall supervision, and reviewed the manuscript. All authors reviewed the manuscript and approved it for submission.

## Funding

This work was supported by the Bill and Melinda Gates Foundation under Grant OPP1156241.

## Conflict of Interest

The authors declare that the research was conducted in the absence of any commercial or financial relationships that could be construed as a potential conflict of interest.

## Publisher's Note

All claims expressed in this article are solely those of the authors and do not necessarily represent those of their affiliated organizations, or those of the publisher, the editors and the reviewers. Any product that may be evaluated in this article, or claim that may be made by its manufacturer, is not guaranteed or endorsed by the publisher.
